# The Design and Experimentation of a Differential Grain Moisture Detection Device for a Combined Harvester

**DOI:** 10.3390/s24144551

**Published:** 2024-07-13

**Authors:** Zheng Liu, Tengxiang Yang, Panpan Li, Jin Wang, Jinshan Xu, Chengqian Jin

**Affiliations:** 1 Nanjing Institute for Agricultural Mechanization, Ministry of Agriculture and Rural Affairs, Nanjing 210014, China; 2 College of Agricultural Engineering and Food Science, Shandong University of Technology, Zibo 255049, China

**Keywords:** grain moisture, capacitive method, differential detection, combined harvester

## Abstract

To conveniently implement the online detection of grain moisture in combined harvesters and the address the influence of the no-load measurement baseline, thereby enhancing detection accuracy and measurement continuity, this study developed a differential grain moisture detection device. For its convenient installation and integration on combined harvesters, a single-pole plate measurement element with a 1.6 mm thick epoxy resin coated with a 2-ounce copper film was designed, and a grain moisture detection device was constructed based on the STM32F103 microprocessor (STMicroelectronics International NV, Geneva, Switzerland). To enhance the device’s interference resistance, a differential amplification measurement circuit integrated with high-frequency excitation was designed using a reference capacitance. To improve the resolution of the measurement circuit, Malab simulations were conducted at different excitation frequencies, ultimately selecting 30 kHz as the system’s excitation signal frequency. To validate the effectiveness of the measurement circuit, validity tests were performed on the constructed sensor, which showed that the sensor’s measurement voltage could effectively distinguish the moisture levels in grains, with a determination coefficient (R²) reaching 0.9978. To address the errors in moisture measurement caused by changes in grain temperature, an interaction experiment of the effect of moisture content and temperature on the measurement voltage was conducted using an integrated temperature sensor, resulting in the construction of a moisture content calculation model. Both the indoor static detection and field testing of the moisture detection device were conducted, indicating that the maximum average error in static measurements was 0.3%, with a maximum relative error of 0.47%, and the average relative error in field tests was ≤0.4%.

## 1. Introduction

When a combine harvester operates at a constant speed, the grain moisture content significantly impacts its feed rate. Higher moisture content generally results in an increased feed rate [1], which in turn raises the load on the threshing and cleaning systems. This can lead to a higher grain breakage rate, reduced threshing efficiency, and increased grain loss, all of which diminish the overall harvest quality [2,3,4,5]. Moreover, a high moisture content can cause issues such as grain conveyance blockages and drum blockages. Therefore, the dynamic adjustment of the operating speed is typically required to maintain a relatively stable feed rate. Hence, the grain moisture content is crucial for both harvest quality and operational safety. Additionally, the real-time monitoring of grain moisture content is crucial for the online yield measurement of harvesters, as it is one of the key factors ensuring the accuracy of yield distribution maps [6,7]. Therefore, developing online detection technologies for the grain moisture content in combined harvesters not only guides the harvester in performing high-quality operations but also effectively realizes precision agricultural management [8,9].

Grain moisture content detection methods are typically classified into direct and indirect measurement techniques. Indirect measurement methods encompass various approaches, including microwave [10,11], near-infrared [12,13], and dielectric constant measurements [14]. The capacitive method based on dielectric properties boasts high reliability, strong adaptability, and excellent dynamic response, making it the widely adopted technique for the online detection of grain moisture content. R. Thakur et al. [15] designed a cylindrical grain moisture detection device with a fixed frequency of 230 kHz based on the principle of capacitive oscillation energy storage, achieving a measurement accuracy of 1% through temperature compensation. In the field of online grain moisture detection for combine harvesters, Chen Jin et al. [16] developed a bipolar plate high-frequency capacitive detection device using a 10 MHz high-frequency excitation source, expanding the measurement range from 10% to 30%, with a maximum relative error of 2.07% in field online monitoring, yet without considering the influence of environmental factors such as temperature. Li Zefeng et al.’s [17] design used the parallel plate capacitance method combined with “fuzzy logic” to address temperature effects, improving temperature adaptability, although this led to significant data fluctuations. However, its complex structure did not accurately reflect the moisture content of the actual harvested grain flow. These studies all implemented moisture inversion through direct frequency fitting but failed to consider the variations in the baseline measurements of the plates under different installation conditions.

To facilitate the online detection of grain moisture content in combine harvesters, address the impact of no-load measurement baseline factors, and enhance detection accuracy and measurement continuity, this study employs a capacitive grain moisture detection principle. Utilizing a differential amplification detection method and incorporating a reference capacitor, we designed a simple monoplate grain moisture content online detection device. This device converts capacitive frequency signals into voltage signals to enable continuous measurement.

## 2. Measurement Principle and Overall Scheme Design

### 2.1. Capacitive Grain Moisture Content Measurement Principle

In physics, the formula for calculating capacitance is

(1) $$C=\frac{\varepsilon_{0}*\varepsilon_{r}*s}{d}$$

where $\varepsilon_{0}$ is the permittivity of free space, $\varepsilon_{0}$ is the relative permittivity of the medium, s is the effective relative area of the capacitor plates, and d is the distance between the capacitor plates.

The principle of using the capacitance method to detect grain moisture content is based on the differing dielectric properties of water, grain, and air [18,19]. Water has the highest dielectric constant, followed by grain, and air has the lowest. Under the fixed structural parameters of the capacitor (*s* and *d*), the relative permittivity $\varepsilon_{r}$ of the capacitor is directly proportional to the moisture content of the grain, resulting in a capacitance value that changes with the moisture content. This change is primarily reflected in the charging and discharging characteristics of the capacitor. Equations (2) and (3) represent the formulas for the *RC* circuit time constant τ and the capacitor charging voltage $U_{c}$ as functions of time, respectively:

(2) τ=R∗C

(3) $$U_{c}=U*[1-e^{-\frac{t}{\tau }}]$$

In the formulas, R,C represent the resistance and capacitance values of the *RC* circuit, respectively, U is the supply voltage, and t is the time variable. As indicated by the formula, the larger the capacitance, the higher the charging voltage of the capacitor under the same charging time. By measuring the electrical characteristics of the formed capacitor, the moisture content of the grain can be indirectly determined.

The relative permittivity $\varepsilon_{r}$ of the medium is generally affected by temperature; typically, higher temperatures result in an increased $\varepsilon_{r}$. Therefore, it is necessary to measure the grain temperature in this design to correct the detected moisture content values.

### 2.2. Scheme Design

#### 2.2.1. Design of the Monitoring System Scheme

**Figure 1:**
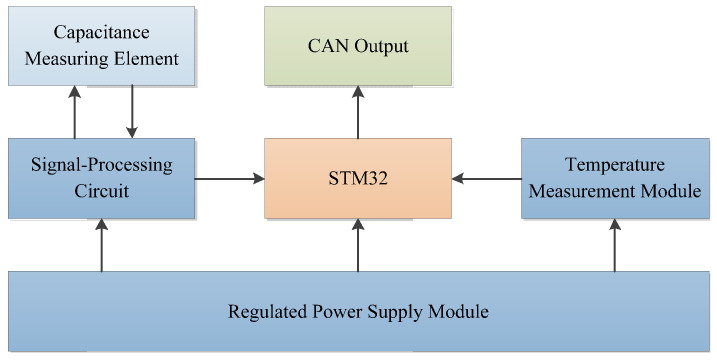
Structural diagram of the detection system design scheme.

The moisture content detection device designed in this study uses STM32F103C8T6 as its processor. The output signal from the capacitive measurement element is converted into a voltage signal via a processing circuit, which is then sent to the processor’s internal A/D converter for analog-to-digital conversion. The grain moisture content is subsequently calculated using an internal moisture content model and is output in real time via the CAN bus. The design scheme of the detection system is shown in Figure 1.

The detection system is powered by an external DC power source through a voltage regulator module. The signal processing circuit consists of a capacitor charge–discharge switching circuit and a signal detection circuit. Upon system startup, the capacitor charge–discharge switching circuit charges and discharges the capacitive measurement element. The capacitance value of the measurement element varies with the grain moisture content, leading to different charge–discharge times and thus varying amplitudes of the output weak electrical signal. This weak signal is then converted into a clean DC voltage signal by the signal detection circuit within the signal-processing circuit, which is subsequently sent to the STM32’s A/D converter for signal processing to obtain the measured voltage. Meanwhile, the STM32 reads the temperature value from the temperature measurement module in real time. Using the internal moisture content calculation model, it calculates the grain moisture content in real time. Finally, the measurements of grain moisture content and temperature are output through the CAN port.

#### 2.2.2. Structural Design of the Detection Device

**Figure 2:**
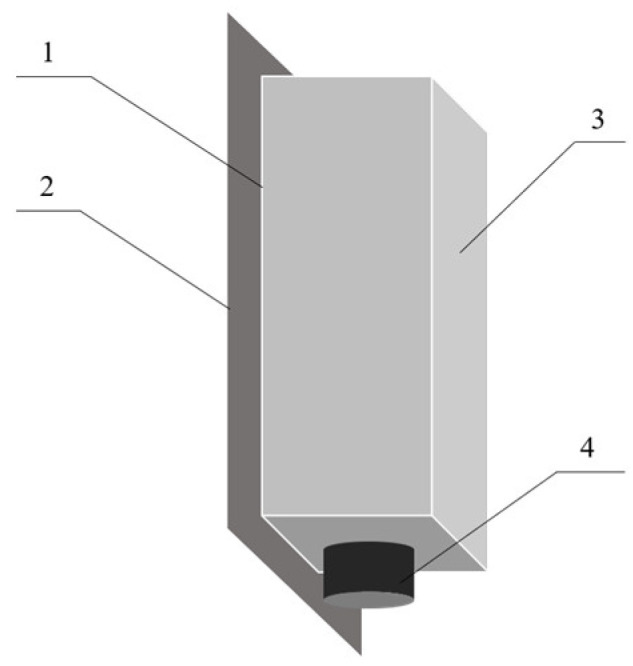
Structural design scheme of the grain moisture content detection device: 1. the temperature sensor, 2. the grounding-protective shield, 3. the sensor casing, 4. the waterproof connector.

References [17,20] involve the design of a cyclic sampling mechanism on the side wall of the elevator of the combine harvester for moisture content sampling and detection, while references [16,21] incorporate a grain-receiving sampling mechanism at the outlet of the grain silo for moisture content sampling and detection. Both methods use sampling techniques for discontinuous detection, resulting in low data continuity. To facilitate the integration of the grain moisture content detection device and achieve continuous measurement, this study selects the bottom of the transverse auger of a combine harvester as the measurement point. Consequently, the structural design of the moisture content detection device is carried out, as shown in Figure 2.

The measurement element employs a single-plate design, with the electrode plate placed inside the device housing. A grounding-protective cover, secured by bolts to the harvester body, ensures easy installation and the reliable grounding of the circuit. The temperature sensor probe is embedded at the bottom of the device to ensure direct contact with the grain during measurement, thereby enabling reliable grain temperature measurement.

### 2.3. Differential Amplification Detection Method

**Figure 3:**
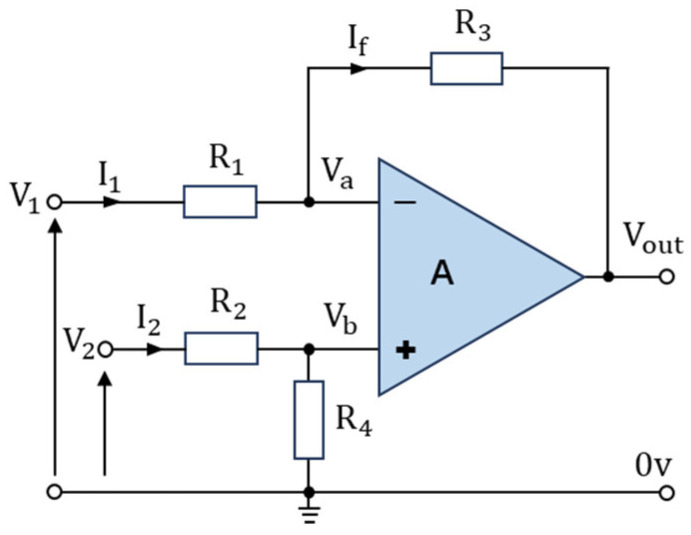
Differential operational amplifier circuit.

In the sensor development or processing of weak low-frequency signals, direct coupling amplification generally exhibits issues such as zero drift and significant interference [22,23]. Due to its excellent electrical symmetry and anti-interference capabilities, differential amplification detection is commonly used in the design and development of sensors and high-speed circuits [24,25,26]. Figure 3 illustrates a differential amplification circuit based on an operational amplifier.

$V_{1}$ and $V_{2}$ are the two independent input signals of the operational amplifier. Interference noise on both signal lines is almost simultaneously coupled. The output is the difference between the two signals, which effectively cancels out common-mode noise. Therefore, this method can effectively suppress the occurrence of zero drift. The formula for the output signal is

(4) $$V_{out}=V_{2}*\frac{R_{4}}{R_{2}+R_{4}}*\frac{R_{1}+R_{3}}{R_{1}}-V_{1}*\frac{R_{3}}{R_{1}}$$

When *R*_1_ = *R*_2_ and *R*_3_ = *R*_4_, the output of the differential operational amplifier can be simplified to the following expression:

(5) $$V_{out}=(V_{2}-V_{1})*\frac{R_{3}}{R_{1}}$$

From Equation (5), it is evident that by “comparing” the two input signals, the differential and amplification of two sets of signals can be accomplished, effectively suppressing common-mode noise in the detection signal. Therefore, this study adopts this method for voltage signal detection and processing in the capacitive moisture content detection device.

## 3. Grain Moisture Content Detection Device Design

### 3.1. Hardware Circuit Design

#### 3.1.1. Measuring Element

**Figure 4:**
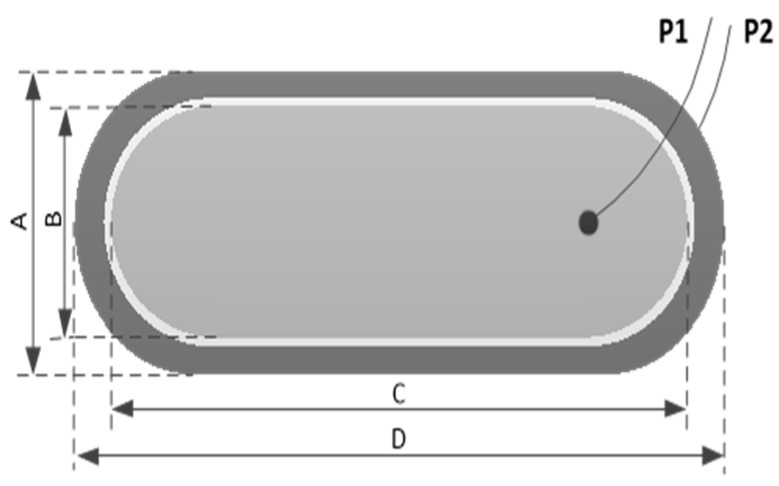
Structure diagram of the measuring element: A. width of polar plate P2, B. width of polar plate P1, C. length of polar plate P1, D. length of polar plate P2.

To meet the compact size requirements for integration into combine harvesters, the measuring element is designed with a 1.6 mm thick epoxy resin covered with a 2-ounce copper film, consisting of two poles, P1 and P2. P1 serves as the positive pole connected to the excitation signal, while P2 is the grounding pole. To enhance the anti-interference capability of the excitation signal, P2 is connected to the edge shielding strip of P1 through vias. The spacing between the shielding strip and the edge of P1 is 0.254 mm. The structure of the measuring element is shown in Figure 4.

The specifications of the measuring plates directly determine the capacitance of the sensor formed, which significantly impacts the dynamic response of the measuring circuit. As indicated by Equations (2) and (3), the larger the capacitance C, the higher the steady-state output voltage amplitude.

Due to spatial constraints, the specification parameters of the measuring element are designed as shown in Table 1. The no-load capacitance value of the measuring element is approximately 8 pF.

#### 3.1.2. Signal Processing Circuit

**Figure 5:**
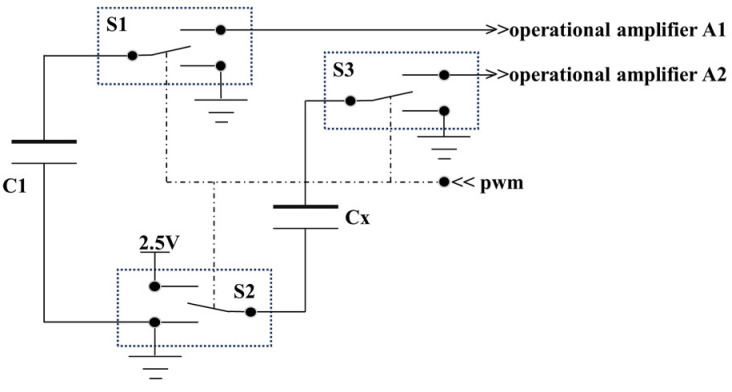
Charging and discharging switch circuit: $S_{1}$, $S_{2}$, and $S_{3}$. the 3-channel high-speed analog switch, pwm. the high speed pulse signal from processor, $C_{1}.$ the adjustable capacitor, $C_{x}$. the capacitor to be measured.

Due to the measuring element’s capacitance of approximately 8 pF, an adjustable capacitor $C_{1}$ with a range of 6 to 10 pF is chosen as the reference capacitor. The circuit utilizes a 3-channel high-speed analog switch chip 74HC4053D (NXP Semiconductors N.V., Eindhoven, The Netherlands) and a 4-channel integrated operational amplifier AD8604ARUZ (Analog Devices, Inc., Norwood City, OH, USA) to construct the charging and discharging switch circuit and the signal processing circuit. The circuit layout is shown in Figure 5. The operating voltages for the chips are DC 2 V to 6 V and DC 2.7 V to 5.5 V, respectively, with the design using a 5 V DC power supply.

**Figure 6:**
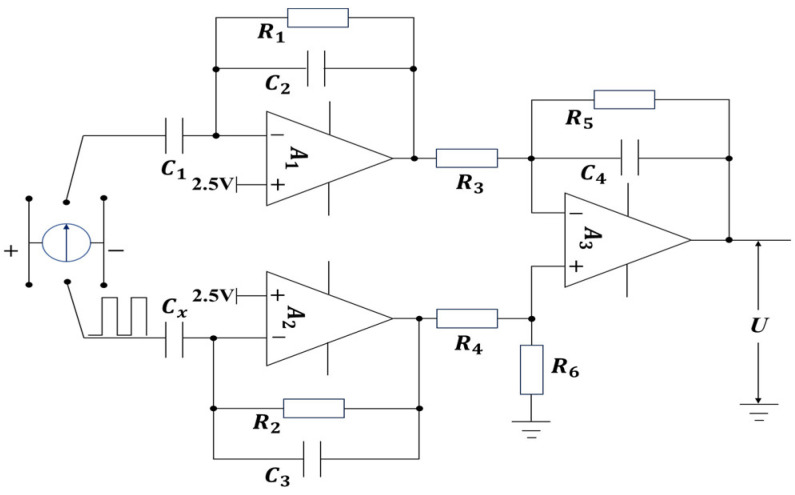
Schematic diagram of the signal-processing circuit: $A_{1}$, $A_{2}$, $A_{3}$. the 3-channels operational amplifier of AD8604ARUZ, $C_{1}.$ the adjustable capacitor, $C_{x}$. the capacitor to be measured, U. the output voltage of the differential circuit.

In the 74HC4053D chip, the three switch channels, $S_{1}\;$, $S_{2}$, and $S_{3}$ are synchronously triggered by the processor’s PWM pulse signal. When the pulse signal is at a low level, the enabled signals of $S_{1}\;$, $S_{2}$, and $S_{3}$ are all grounded, and both poles of $C_{1}$ and $C_{x}$ are grounded to discharge. When the pulse signal is at a high level, $S_{1}$ is enabled and connected to operational amplifier $A_{1}$, one pole of $S_{2}$ is connected to a 2.5 V power supply (half of the working voltage of AD8604ARUZ), and the other pole is enabled and connected to operational amplifier $A_{2}$, charging and signal detection are performed on $C_{1}$ and $C_{x}$. The signal processing circuit built based on AD8604ARUZ is shown in Figure 6.

The operational amplifier channels $A_{1}$ and $A_{2}$ use circuits with identical resistors and capacitors to construct two capacitive charging and discharging amplification circuits. During measurement, the charges accumulated at the terminals of $C_{1}$ and $C_{x}$ are proportionally amplified, producing two signal voltages, $U_{o1}$ and $U_{o2}$, as calculated in Equations (4) and (5).

(6) $$U_{o1}=-A*(U_{in1}+U_{f1})$$

(7) $$U_{o2}=-A*(U_{in2}+U_{f2})$$

The feedback networks of operational amplifiers $A_{1}$ and $A_{2}$ are negative feedback networks composed of resistors and capacitors, with the corresponding feedback voltages being

(8) $$U_{in1}=2.5-V_{c1}$$

(9) $$U_{in2}=2.5-V_{cx}$$

(10) $$U_{f1}={(U}_{o1}-V_{c1})*\frac{R_{1}}{R_{1}+\frac{1}{jwC_{2}}}$$

(11) $$U_{f2}={(U}_{o2}-V_{cx})*\frac{R_{2}}{R_{2}+\frac{1}{jwC_{3}}}$$

Since the operational amplifiers $A_{1}$ and $A_{2}$ operate under negative feedback, their closed-loop gain is significantly lower than their open-loop gain. Therefore, it can be approximated that their output voltage is only related to the input voltage and the feedback network, and independent of the specific value of *A*. By substituting Equations (8)–(11) into Equations (6) and (7), it can be derived that:

(12) $$U_{o1}=V_{c1}-\frac{2.5(1+jwR_{1}C_{2})}{1+2jwR_{1}C_{2}}$$

(13) $$U_{o2}=V_{cx}-\frac{2.5(1+jwR_{2}C_{3})}{1+2jwR_{2}C_{3}}$$

$U_{o1}$ and $U_{o2}$ are fed into a differential circuit composed of $A_{3}$ for processing, then the output voltage is calculated as shown in Equation (14).

(14) $$U=\frac{\left(R_{3}{+R}_{5}\right)*R_{6}}{\left(R_{4}{+R}_{6}\right)*R_{3}}U_{o2}-\frac{R_{5}}{R_{3}}U_{o1}$$

Using a symmetrical circuit design, where $R_{1}=R_{2},R_{3}=R_{4},R_{5}=R_{6},C_{2}=C_{3}$, by substituting Equations (4) and (5) into Equation (14), the output voltage of the differential circuit can be obtained:

(15) $$U=\frac{R_{5}}{R_{3}}(V_{cx}-V_{c1})$$

Since $V_{c1}$ is constant, the output voltage of the measuring circuit is determined by the size of the capacitor being tested. The larger the capacitor, the higher its steady-state voltage, and consequently, the higher the measuring voltage of the circuit.

#### 3.1.3. Frequency Response Analysis of Incentive Signals

The switching frequency directly affects the output characteristics of the measurement circuit. To determine the appropriate incentive signal frequency, multiple incentive frequencies ranging from 1 kHz to 100 kHz were selected using MATLAB (version: R2022b 9.13.0.2049777) to perform simulation analysis on the signal processing circuit shown in Figure 6. Both the $C_{1}$ and $C_{x}$ values used in the simulation were 5 pF. The simulation results are shown in Table 2.

**Figure 7:**
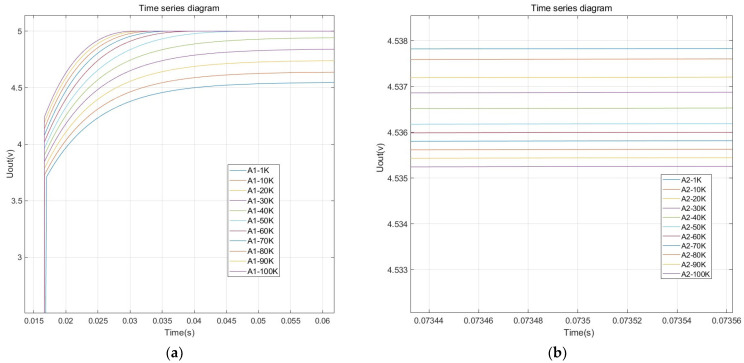
Measurement circuit simulation results: (**a**) the $A_{1}$ output frequency response; (**b**) the $A_{2}$ output frequency response.

The frequency response curve is shown in Figure 7.

From the simulation results, it can be seen that the steady-state output voltage of $A_{1}$ increases with the increase in the incentive signal frequency, and when the frequency exceeds 40 KHz, the output stabilizes at 5 V. On the other hand, the steady-state output voltage of $A_{2}$ decreases with the increase in the incentive signal frequency. To ensure that the measurement circuit operates in a non-saturated state with high resolution, 30 kHz is selected as the system’s incentive signal frequency.

#### 3.1.4. Temperature Measurement Module

The temperature sensor used is the through-hole DS18b20. The sensor operates at a DC voltage of 3.3 V and has a temperature detection range of −10 °C to 85 °C. The measurement error is less than ±0.5%, which meets the temperature measurement requirements of the harvesting environment.

#### 3.1.5. Regulated Power Supply Module

The detection device is powered by a 12 V DC vehicle power supply. To supply power to the processor, detection circuit, and communication circuit, the L78M05ABDT and AMS1117-3.3 chips are used to provide 5 V and 3.3 V operating voltages, respectively. To provide a stable 2.5 V forward input signal to channels $A_{1}$ and $A_{2}$ of the AD8604ARUZ integrated operational amplifier shown in Figure 6, the LM4040BIM3-2.5 precision micropower voltage reference chip is used to generate a 2.5 V power signal.

### 3.2. Software Design

**Figure 8:**
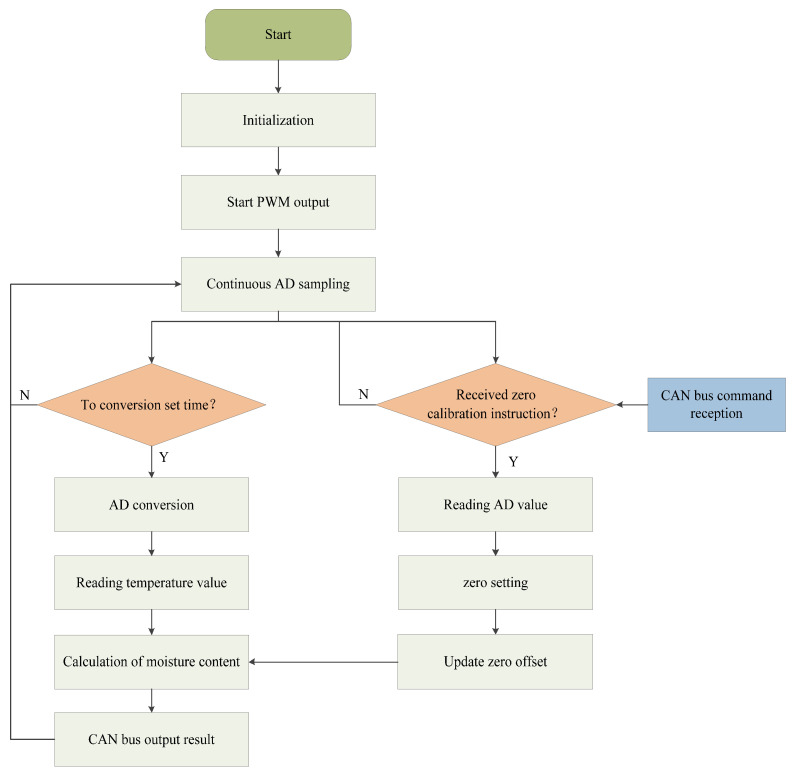
Software flowchart.

After the detection device is started, a timer generates a 30 kHz high-speed pulse signal to control the 74HC4053D high-speed switch, which performs the charging and discharging excitation of the measurement element. The circuit output voltage signal is continuously sampled by the STM32 internal AD, with a sampling frequency of 47.6 kHz. The program samples multiple times and performs software filtering and an average calculation every second. It synchronously reads the temperature and calculates the grain moisture content based on the dynamic measurement model of grain moisture content. The measurement results are output through the CAN bus. The measurement program flow is shown in Figure 8.

The program design introduces a “no-load zero calibration” mechanism. After the device is installed, a zero calibration command is sent via CAN communication before formal measurement. The software automatically performs bias calibration to eliminate system errors caused by zero point offset due to the installation structure. This improves the device’s adaptability to different installation environments and enhances measurement accuracy.

## 4. Grain Moisture Detection Device Calibration Test

### 4.1. Sensor Effectiveness Test

To verify whether the sensor can effectively reflect the grain moisture content levels, a validity test of the sensor was conducted. Under room temperature conditions, dried soybean samples were placed in a material box, and a plasma water mist was sprayed onto the samples using a sprayer. After each spray, the samples were thoroughly mixed from top to bottom and allowed to stand for 0.5 h. This process was repeated three times. After spraying and thoroughly mixing, sufficient samples were sealed in bags. This procedure was repeated until eight sets of samples were prepared. At room temperature, the detection device was used to measure the voltage (*U*) of the eight sample sets. Subsequently, the standard moisture content was determined using an electric blast drying oven, following the standard drying method at 105 °C [27]. The main parameters of the electric blast drying oven are listed in Table 3.

The standard moisture content (*M*) was calculated using the mass difference of the samples before and after drying, measured with a precision balance. The calculation method is shown in Equation (16):

(16) $$M=\frac{m_{1}-m_{2}}{m_{1}}\times 100\%$$

where $m_{1}$ is the mass of the sample before drying, and $m_{2}$ is the mass of the sample after drying. The experimental data are shown in Table 4.

**Figure 9:**
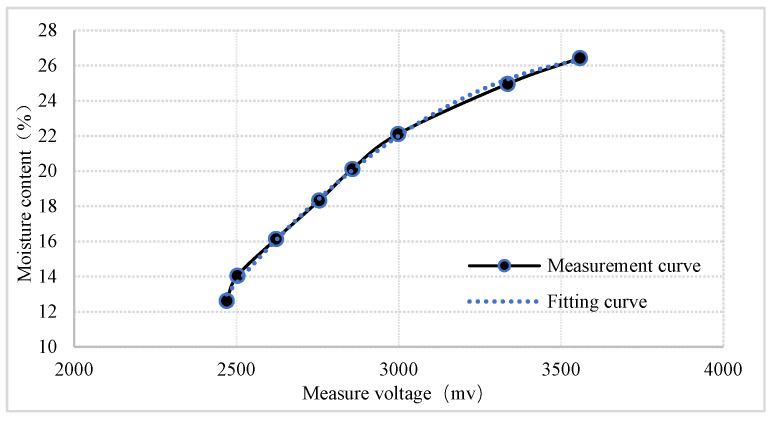
Nonlinear fitting curve between the measured voltage and the standard moisture content.

From Table 4, it can be seen that the sensor measurement voltage shows good monotonic consistency with the soybean moisture content. The measurement data were subjected to nonlinear fitting, as shown in Figure 9.

The relationship between the sensor measurement voltage and the soybean moisture content obtained from the fitting is

(17) $$M=-0.000009U^{2}+0.064U-92.665$$

In the equation, M represents the grain moisture content in a percentage (%), and U represents the measurement voltage in millivolts (mv). The coefficient of determination, R^2^ = 0.9978, indicates that the designed sensor can effectively distinguish the moisture content levels of the grain.

### 4.2. Dynamic Moisture Content Measurement Model

Establishing an accurate mathematical model for moisture content detection is key to achieving high-precision moisture content measurements. During the grain moisture measurement process, changes in grain temperature can cause variations in the dielectric constant, thereby introducing measurement errors [28]. To eliminate this error, the literature [29,30] employed segmented and linear compensation methods for error correction. However, these methods struggle to address the temperature effects across different types of grains and a wide measurement range.

#### 4.2.1. Experiment Preparation

In this design, there is a coupling relationship among the true moisture content of the grain, the measured voltage, and the sample temperature. To construct an accurate soybean moisture content measurement model, experimental research was conducted using high-quality soybeans from Northeast China harvested in 2023. The experiments employed a Huamai SPX constant temperature and humidity test chamber (Shaoxing Huamai Instrument Manufacturing Co., Ltd, Shaoxing, China) to adjust the sample temperature and the experimental environment’s temperature and humidity. The main parameters are listed in Table 5.

**Figure 10:**
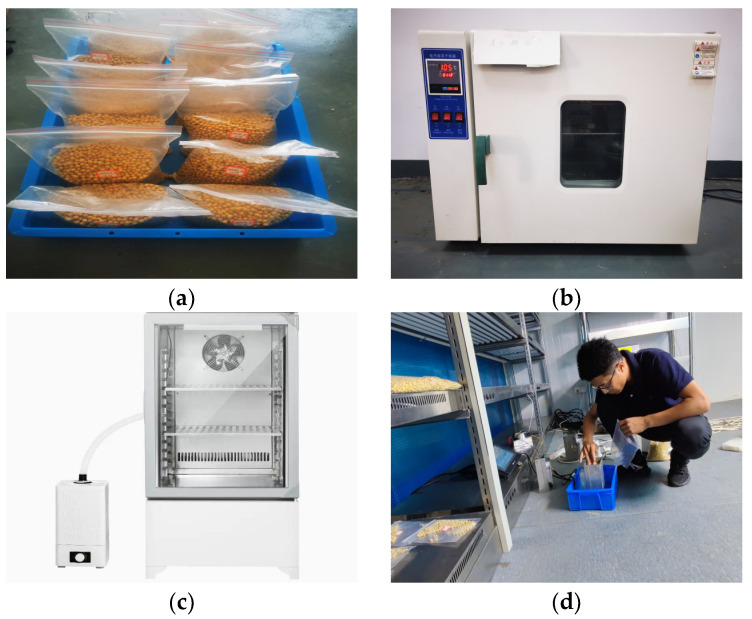
Charging and discharging switch circuit: (**a**) test sample; (**b**) 105 °C drying; (**c**) constant temperature and humidity test chamber; (**d**) device measurement.

Typically, when soybeans are harvested, the ambient temperature ranges from 15 °C to 30 °C, and the soybean moisture content is within the 15% to 25% range [31]. For the experiment, the temperature range was set from 15 °C to 35 °C, with the humidity was maintained at approximately 50%. The experimental process is shown in Figure 10.

#### 4.2.2. Construction of the Moisture Content Calculation Model

Referencing Section 4.1, prepare eight sealed samples with different moisture content levels. From each of these eight samples, take an appropriate amount to determine the standard moisture content using the 105 °C standard drying method. Place the ten sealed samples simultaneously into the constant temperature and humidity test chamber. Starting from 15 °C, use the detection device to read the measurement voltage and measurement temperature (*T*) for all samples. After each round of measurement, increase the temperature by 3 °C. Conduct the next round of measurements after the temperature stabilizes for 0.5 h at the set temperature, continuing this process until the temperature exceeds 35 °C. Some experimental data records are shown in Table 6.

**Figure 11:**
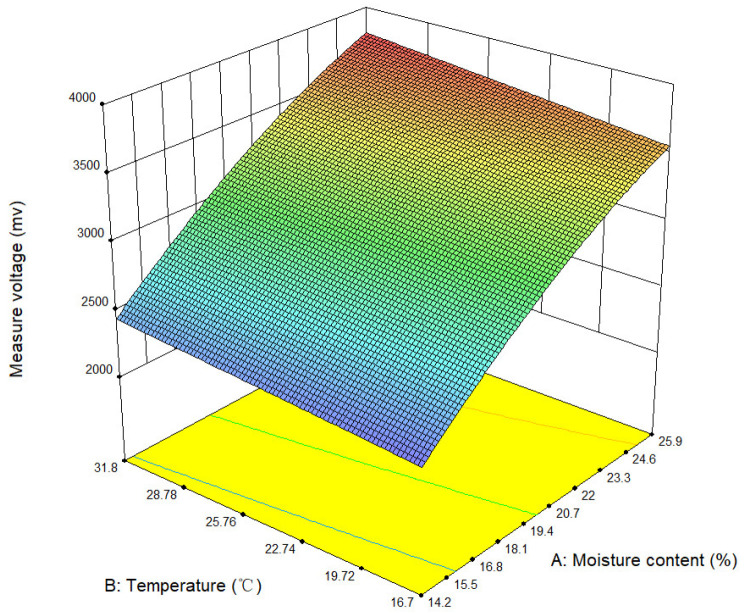
A 3D diagram of the interaction effect of moisture content and temperature on measurement voltage.

Based on the experimental data in Table 6, the influence of moisture content and temperature on the device’s measured voltage was analyzed using Design Expert (version: 10.0.4.0). The interaction effect of actual soybean moisture content and sample temperature on the device’s measured voltage was established, as shown in Figure 11.

As shown in Table 6 and Figure 11, two key observations can be made: First, at a constant temperature, the higher the actual moisture content of the soybeans, the higher the measured voltage of the detection device. Second, for soybean samples with constant moisture content levels, the higher the sample temperature, the higher the measured voltage of the detection device. Therefore, the measured voltage signal of the detection device is positively correlated with both the soybean moisture content and temperature. Accordingly, a regression model for soybean moisture content was constructed based on Table 6. This model enables the calculation of the actual moisture content of soybeans using the measured voltage and temperature from the detection device. The regression model is established as follows:

(18) $$M=33.71731+0.0461T-\surd (906.76316-{0.01152T}^{2}+5.5777T-0.26067U)$$

In the variance analysis of regression model (18), the Prob > F value for the model and the moisture content factor is less than 0.0001, and the Prob > F value for the temperature factor is 0.0002. This indicates that the regression model is highly significant, and the effects of moisture content and temperature on the measurement voltage are extremely significant.

## 5. Experiment

### 5.1. Static Test of Grain Moisture Content Detection

Under room temperature conditions, refer to Section 4.1 to prepare six sealed samples with different moisture content levels. The moisture content was measured using both the grain moisture content detection device and the 105 °C drying method. The moisture contents measured by the drying method were 12.71%, 14.58%, 16.32%, 18.19%, 20.43%, and 23.25%. The grain moisture content detection device was used to measure the moisture content of the samples in an environment with a room temperature of 18 °C and a humidity of 37% RH. Each sample was measured three times, and the average value was taken. The experimental data are shown in Table 7.

From Table 7, it can be observed that within the moisture content range of 12% to 23%, the device’s maximum average static measurement error is 0.30%, and the maximum relative error is 0.47%. The measurement results are relatively stable. When the moisture content is lower, the maximum relative error is relatively larger; when the moisture content is higher, the maximum error is relatively smaller. As the moisture content level increases, the maximum relative error tends to decrease, indicating that the device’s measurement performance improves.

The main reasons for the measurement errors mentioned above may include the following three points:

(1) The experiment was conducted in an open indoor environment, where fluctuations in the ambient temperature during the test could affect the results.

(2) The airflow’s impact on the samples caused moisture evaporation, leading to changes in the actual moisture content of the samples.

(3) Repeatedly pouring the samples during the experiment accelerated moisture evaporation, and some moisture was absorbed by the walls of the measurement container, resulting in changes in the actual moisture content of the samples during the measurement.

### 5.2. Field Test of Grain Moisture Content Detection

**Figure 12:**
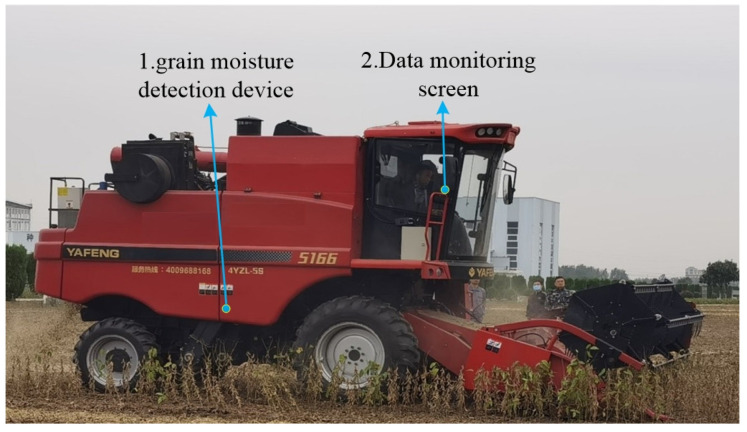
Field experiment diagram.

To verify the reliability of the device, a moisture content detection test was conducted on soybean harvesting using a Yafeng 5166 soybean harvester (Shandong Yafeng Agricultural Machinery Equipment Co., Ltd., Zibo, China). The soybean variety tested was Zhonghuang 37. As shown in Figure 12, the moisture content detection device was installed at the bottom of the transverse auger of the soybean harvester, ensuring full contact between the device and the soybean grains during harvesting. The display screen was installed in the cabin for data viewing and storage. During the experiment, the sensor data output frequency was set to 1 Hz. Every 10 s, the harvester was stopped once to record 10 sets of data, and 3 sets of samples were taken from the grain bin and sealed in bags for the subsequent testing of the true moisture content of the soybeans using the 105 °C drying method. The experiment was repeated six times, and the results are shown in Table 8.

Field test results showed that the average measurement error of soybean moisture was ≤0.4%, indicating that the designed differential grain moisture detection device is suitable for online moisture content determination during soybean mechanical harvesting.

## 6. Conclusions

(1) Based on the characteristics of capacitor charging and discharging, this paper employs a differential amplification detection method to design a differential grain moisture measurement circuit that integrates moisture detection, temperature compensation, and online moisture content calculation. The frequency response characteristics of the measurement circuit were simulated using MATLAB. The results indicated that when the excitation signal frequency was 30 kHz, the resolution of the circuit’s measurement signal was relatively high. The effectiveness of the sensor was tested, and the results showed that the output voltage of the measurement circuit had good monotonic consistency with the moisture content of soybeans, effectively reflecting the grain moisture level.

(2) For grain samples with different moisture content and temperature levels, Design Expert was used to analyze the influence patterns of grain moisture content, grain temperature, and device detection voltage signals. The results showed that the voltage signal of the measurement circuit was positively correlated with both grain moisture content and temperature. The constructed mathematical model of grain moisture content effectively reduced the measurement error caused by temperature factors.

(3) The developed grain moisture detection device was validated through both indoor static tests and field tests. The maximum average error of static measurements was 0.3%, with a maximum relative error of 0.47%. The average relative error in field tests was less than or equal to 0.4%.

The differential grain moisture content detection device designed in this study improves the accuracy of grain moisture detection and demonstrates good consistency and real-time performance. Our next steps include continuing experimental research and developing measurement devices for moisture content models in various crops such as rice, wheat, and corn. Additionally, we aim to integrate online grain moisture detection technology with yield monitoring in combine harvesters. By determining the grain moisture content in real time, we can calculate the real-time harvest yield and achieve the dynamic monitoring of yield distribution using high-precision satellite positioning. Furthermore, in terms of the intelligent regulation of harvester operating parameters, we plan to predict the feed rate of the harvester by detecting grain moisture content, header height, and operating speed, thereby adjusting the vehicle’s travel speed to ensure safe and high-quality operations.

## Figures and Tables

**Table 1:** Specification parameters of the measuring element.

Specifications of Measurement Elements (mm)				Capacitance Value (pF)
A	B	C	D	
30	24	56	60	about 8

**Table 2:** Frequency characteristic simulation results.

Serial Number	Frequency (KHz)	$A_{1}$ Output (V)	$A_{2}$ Output (V)
1	1	4.54203	4.53744
2	10	4.63359	4.53716
3	20	4.73478	4.53675
4	30	4.83629	4.53642
5	40	4.93757	4.53607
6	50	4.99967	4.53573
7	60	4.99968	4.53554
8	70	4.99968	4.53536
9	80	4.99968	4.53517
10	90	4.99969	4.53499
11	100	4.99969	4.53478

**Table 3:** Main parameters of the drying oven.

Serial Number	Parameter	Specification
1	Internal Dimensions	450 × 350 × 450 mm
2	Power	2000 W
3	Temperature Range	10 °C~300 °C
4	Temperature Accuracy	1 °C

**Table 4:** Sensor validity test data.

No	M (%)	U (mV)
1	12.62	2470
2	14.05	2503
3	16.14	2622
4	18.33	2755
5	20.12	2857
6	22.11	2998
7	24.96	3335
8	26.43	3558

**Table 5:** Main parameters of the SPX constant temperature and humidity test chamber.

Serial Number	Parameter	Specification
1	Internal Dimensions	500 × 400 × 750 mm
2	Power	900 W
3	Humidity Range	20.0~99.9%
4	Humidity Resolution	0.1%
5	Temperature Range	0 °C~85 °C
6	Temperature Resolution	0.1 °C

**Table 6:** Measurement data at different moisture content and temperature levels.

No	M (%)	T (°C)	U (mV)
1	14.17	16.7	2268
2	15.33	16.9	2411
3	17.04	17.3	2655
4	18.87	17.8	2901
5	20.51	18.4	3010
6	22.02	18.7	3258
7	24.18	18.9	3450
8	25.93	19.0	3580
9	14.17	25.9	2341
10	15.33	25.8	2530
11	17.04	25.6	2767
12	18.87	25.3	3004
13	20.51	24.8	3113
14	22.02	24.5	3348
15	24.18	24.4	3543
16	25.93	24.8	3659
17	14.17	31.8	2420
18	15.33	31.6	2603
19	17.04	31.5	2842
20	18.87	31.3	3080
21	20.51	30.5	3251
22	22.02	30.0	3411
23	24.18	29.8	3603
24	25.93	29.8	3793

**Table 7:** Verification test data of grain moisture content device.

No	Moisture Content (%)		Average Relative Error (%)	Maximum Relative Error (%)
	Drying Method Measurement Value	Device Measurement Average Value		
1	12.71	12.65	0.29	0.47
2	14.58	14.56	0.30	0.41
3	16.32	16.33	0.12	0.18
4	18.19	18.18	0.11	0.16
5	20.43	20.45	0.08	0.15
6	23.25	23.22	0.10	0.13

**Table 8:** Results of field experiments.

NO	Moisture Content (%)		Average Relative Error (%)
	Average Value of Drying Method Measurement	Average Value of Device Measurement	
1	14.82	14.76	0.40
2	14.77	14.72	0.34
3	15.36	15.40	0.26
4	15.13	15.08	0.33
5	16.20	16.24	0.25
6	15.83	15.79	0.32

## Data Availability

Data are contained within the article.
